# Paravertebral Block for Multiple Rib Fractures in an Anticoagulated Trauma Patient

**DOI:** 10.7759/cureus.61834

**Published:** 2024-06-06

**Authors:** Malcolm Lee, Michael Ayad, Jose L Diz Ferre, Lori Ann Oliver, Sabry Ayad

**Affiliations:** 1 Outcomes Research, Ohio University Heritage College of Osteopathic Medicine, Cleveland, USA; 2 Outcomes Research, Lake Erie College of Osteopathic Medicine, Cleveland, USA; 3 Outcomes Research, Cleveland Clinic, Cleveland, USA; 4 Anesthesiology, Cleveland Clinic Fairview Hospital, Cleveland, USA; 5 Outcomes Research and Anesthesiology, Cleveland Clinic, Cleveland, USA

**Keywords:** trauma patient, alcohol use disorder (aud), direct oral anticoagulant therapy, multiple rib fractures, paravertebral block (pvb)

## Abstract

This case report presents the complex analgesia management of a 52-year-old male with a significant medical history including atrial fibrillation treated with apixaban, essential trigeminal neuralgia, non-ischemic cardiomyopathy, and chronic systolic heart failure. The patient experienced a loss of control while riding a motorized bicycle, resulting in a fall and head injury with no loss of consciousness. Upon admission, he tested positive for ethanol, cannabinoids, and oxycodone. The physical exam was significant for right cephalohematoma and right elbow hematoma. Imaging revealed multiple injuries, including right rib fractures (T3-12) with hemothorax. Right paravertebral catheters were placed in the intensive care unit (ICU).

## Introduction

The use of oral direct Xa inhibitors (-xabans) has become more popular due to their effectiveness in preventing ischemic stroke in high-risk patients with atrial fibrillation and prothrombotic or hyper-coagulable states as well as managing deep venous thrombosis and pulmonary embolism. However, rapid reversal of this drug class has proven more challenging due to the requirement of coagulation factors, especially in patients with severe hemorrhage following acute trauma and/or those requiring emergency surgeries or invasive procedures [1].​ The use of apixaban in this case increases the risk of bleeding during invasive procedures including regional anesthesia.

Reversing the anticoagulated state before performing paravertebral blocks is crucial to minimizing the risk of bleeding complications, particularly hematoma formation, which can lead to serious consequences such as nerve injury, spinal cord compression, or even death [2]. Other regional techniques are available, including the erector spinae plane (ESP) blocks, which is known to carry less risk in anticoagulated patients.

However, paravertebral block (PVB) was chosen due to its superior analgesic efficacy for extensive rib fractures and the potential for better respiratory function preservation, which was critical, given the patient's multiple thoracic injuries and small hemothorax [3]. PVBs cover somatic and sympathetic nerves leading to both somatic and visceral pain relief. The decision was made after careful evaluation of the risks and benefits and taking into consideration the availability of coagulation factor reversal agents to mitigate bleeding risks.

## Case presentation

A 52-year-old male with a past medical history significant for hypertension, chronic heart failure with an ejection fraction of 25%, atrial fibrillation on direct Xa inhibitors, and ethanol (ETOH) abuse presented to the emergency department (ED) after falling from a motorized bicycle and sustaining a head injury with no loss of consciousness. His primary complaint was right chest pain on inspiration, with pain scores of 7/10 at rest and 10/10 with ambulation, which was initially treated with oxycodone 10 mg, fentanyl 50 mcg every two hours, and topical lidocaine patch as needed.

**Figure 1:**
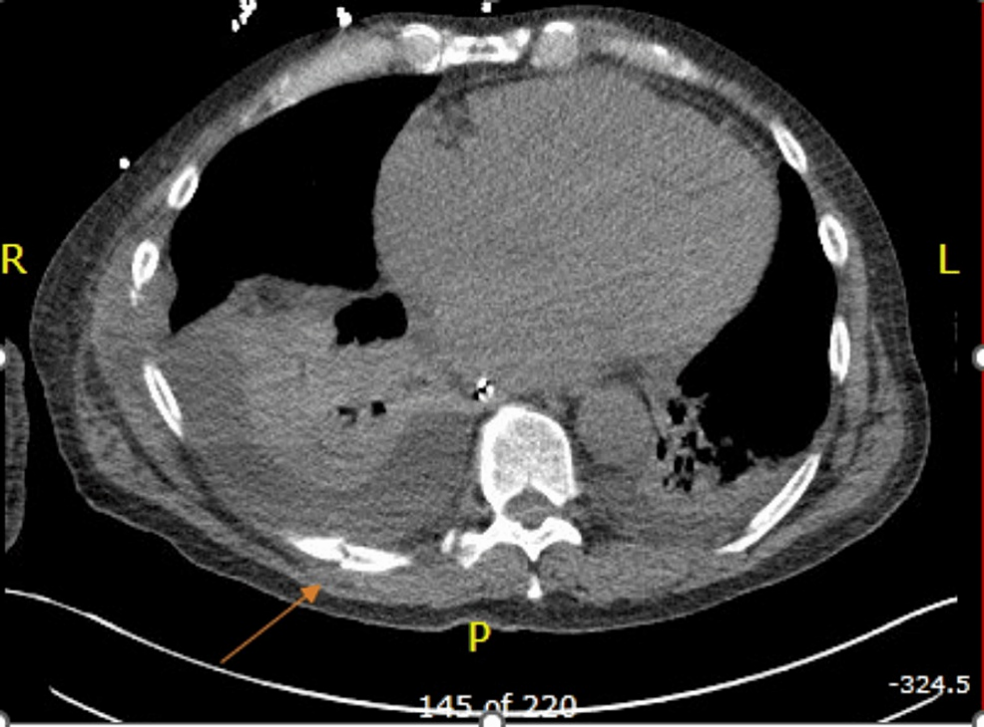
CT chest without contrast CT chest showing right acute rib fracture and small right pleural effusion.

Upon admission, the patient tested positive for ETOH (157 mg/dL), cannabinoids, and oxycodone. Lab results showed sodium 135 mEq/L, potassium 3.3 mEq/L, aspartate aminotransferase 153 U/L, and alanine aminotransferase 110 U/L. The physical exam was significant for right cephalohematoma and a right elbow hematoma. Imaging revealed multiple injuries, including right rib fractures (T3-12) with hemothorax (Figure 1). Additionally, he had an acute right frontal intraparenchymal hemorrhage, a small right frontal subgaleal hematoma with local mass effect, mild perihepatic free fluid, right suprarenal fossa edema, grade-1 right adrenal injury, and grade-1 right diaphragmatic crus injury

The patient was started on an anti-inhibitor coagulant complex for the reversal of apixaban in the ED due to these findings. Trauma surgery was consulted, and requested that the patient be transferred to the intensive care unit (ICU). While admitted, the patient was followed by neurosurgery for his traumatic brain injury (TBI), which was managed conservatively. Repeat imaging remained stable. His right elbow hematoma was managed conservatively. Although thoracic surgery was consulted for possible rib plating, the patient declined. His rib fractures were managed with aggressive bronchopulmonary hygiene and PVBs included in the previous multimodal pain control regimen.

On day 2, right PVB catheters were placed by acute pain medical services (APMS) in the ICU. Lab results at the time showed PT 10.5 sec, PT/INR 0.9, hemoglobin 14.7 g/dL, hematocrit 44.3%, and platelet count 197 k/uL [4]. The procedure was performed at the right levels T4 and T7 with an in-plane parasagittal view using an 18-G Tuohy needle with a length of 90 mm. Saline was used for hydrodissection during 20-G catheters' placement at a depth of 11 cm. The injection assessment yielded negative aspiration without paresthesia. Intermittent bolus of ropivacaine 0.2% 6 mL every 30 minutes with no basal rate was started along with gabapentin 300 mg every 8 hours, intravenous fentanyl 50 mcg every 2 hours, lidocaine patches and oxycodone 5-10 mg every 4 hours as needed. 

The patient reported excellent pain control in the following days. On day 3, 5000 units of subcutaneous heparin were started every eight hours, and the catheters were removed on day 6 by APMS without any signs or symptoms of infection, such as erythema, tenderness, drainage, or warmth. The patient had a prolonged hospital course due to multiple injuries and was discharged on day 25 [5].

## Discussion

ESP, SAP, and PVB are all effective in reducing cumulative 24-hour dynamic pain scores and PVB is an alternative to neuraxial blockade for management of rib fracture pain as it provides unilateral blockade with excellent pain control [6,7]. It reduces the risks of motor weakness, difficult ambulation and urinary retention that are often associated with epidural blockade. PVB is a reasonable option in cases where placement of an epidural catheter is difficult and/or contraindicated, particularly in the case of patients with pre-existing coagulopathy including those with thrombocytopenia or on anticoagulant therapy.

Our patient had a severe intracranial hematoma that required immediate reversal of apixaban, which also facilitated placement of PVB catheters. Compared to warfarin, the use of direct Xa inhibitors has become more popular due to superior prevention of thromboembolism such as deep vein thrombosis, pulmonary embolism in high-risk patients with atrial fibrillation, prothrombotic states, hypercoagulability, or cardiac stent placement [8]. However, the reversal of its anticoagulant effects is more challenging due to the requirement of coagulation factors [1]. This must be considered especially for patients arriving at the emergency setting after acute trauma accidents.

## Conclusions

This case study highlights the significance of anticoagulant therapy management, especially for individuals with complicated medical histories, to successfully undergo regional anesthetic procedures such as PVB catheters. Given the risk of bleeding complications associated with anticoagulant use, especially in emergency settings following traumatic injuries, a careful assessment of the patient's clinical status and rapid reversal of anticoagulated states are imperative.
